# Modulation of 5-fluorouracil as adjuvant systemic chemotherapy in colorectal cancer: the IGCS-COL multicentre, randomised, phase III study

**DOI:** 10.1038/sj.bjc.6602800

**Published:** 2005-10-11

**Authors:** S De Placido, M Lopez, C Carlomagno, G Paoletti, S Palazzo, L Manzione, C Iannace, G P Ianniello, F De Vita, C Ficorella, A Farris, G Pistillucci, M Gemini, E Cortesi, V Adamo, N Gebbia, S Palmeri, C Gallo, F Perrone, G Persico, A R Bianco

**Affiliations:** 1 Dipartimento di Endocrinologia ed Oncologia Molecolare e Clinica, Università Federico II, Napoli, Italy; 2 Struttura Complessa, Oncologia Medica B, Istituto Nazionale Tumori Regina Elena, Roma, Italy; 3 Divisione di Oncologia, Ospedale Mariano Santo, Cosenza, Italy; 4 U.O. di Oncologia Medica, Ospedale San Carlo, Potenza, Italy; 5 Divisione di Chirurgia Generale, Ospedale Moscati, Avellino, Italy; 6 U.O. di Oncologia, ASL-1, Benevento, Italy; 7 Divisione di Oncologia Medica, Seconda Università, Napoli, Italy; 8 Divisione di Oncologia Medica, Università de L'Aquila, Italy; 9 Divisione di Oncologia Medica, Università di Sassari, Italy; 10 Divisione di Oncologia, Ospedale S. Maria Goretti, Latina, Italy; 11 U.O. di Oncologia, Ospedale Belcolle, Viterbo, Italy; 12 U.O. Complessa Oncologia ‘B’ Università La Sapienza, Roma, Italy; 13 Dipartimento di Oncologia medica, A.O.U. Policlinico, Messina, Italy; 14 U.O. di Oncologia, Dipartimento di Oncologia Clinica, Università di Palermo, Italy; 15 Cattedra di Oncologia, Dipartimento di Oncologia Clinica, Università di Palermo, Italy; 16 Dipartimento di Medicina Pubblica, Clinica e Preventiva, Seconda Università, Napoli, Italy; 17 Unità Operativa Sperimentazioni Cliniche, Istituto Nazionale Tumori, Napoli, Italy; 18 Dipartimento Universitario di Chirurgia Generale, Geriatrica, Oncologica e Tecnologie Avanzate, Università Federico II, Napoli, Italy

**Keywords:** adjuvant chemotherapy, colorectal cancer, 5-fluorouracil modulation

## Abstract

The aims of this multicentre, randomised phase III trial were to evaluate: (1) the role of levamisol (LEV); and (2) the role of folinic acid (FA), added to 5-fluorouracil (5FU) in the adjuvant treatment of colorectal cancer. Patients with histologically proven, radically resected stage II or III colon or rectal cancer were eligible. The study had a 2 × 2 factorial design with four treatment arms: (a) 5FU alone, (b) 5FU+LEV, (c) 5FU+FA, (d) 5FU+LEV+FA, and two planned comparisons, testing the role of LEV and of FA, respectively. From March 1991, to September 1998, 1327 patients were randomised. None of the two comparisons resulted in a significant disease-free (DFS) or overall (OAS) survival advantage. The hazard ratio (HR) of relapse was 0.89 (95% confidence intervals (CI): 0.73–1.09) for patients receiving FA and 0.99 (95% CI 0.80–1.21) for those receiving LEV; corresponding HRs of death were 1.02 (95% CI: 0.80–1.30) and 0.94 (95% CI 0.73–1.20). Nonhaematological toxicity (all grade vomiting, diarrhoea, mucositis, congiuntivitis, skin, fever and fatigue) was significantly worse with FA, while all other toxicities were similar. In the present trial, there was no evidence that the addition of FA or LEV significantly prolongs DFS and OAS of radically resected colorectal cancer patients.

Colorectal cancer is the third most frequent cause of death for neoplasm in Western countries. After radical resection, about 30% of stage II and 50% of stage III patients are expected to develop recurrent disease, mostly during the first 5 years after surgery. Adjuvant treatment has been demonstrated to prolong disease-free (DFS) and overall survival (OAS).

When the present trial was planned, the efficacy of adjuvant chemotherapy in prolonging DFS and OAS of colon and rectal cancer patients had been just demonstrated, but the best regimen was far to be identified.

Mostly based on the results of the Intergroup-0035 trial (Laurie *et al*, 1989; Moertel *et al* 1990), that found a survival advantage for the combination 5-fluorouracil (5FU)+levamisol (LEV) as compared to an observation arm, the National Cancer Institute (NCI) Consensus Conference, in 1990, adopted 5FU+LEV as the standard therapy for patients with resected stage III colon cancer (NIH Consensus Conference, 1990). The NCI Consensus Conference confirmed postoperative pelvic irradiation plus 5FU-based regimens to be the standard treatment for stages II and III rectal cancer, as indicated by the results of many published studies (GITSG, 1985; Krook *et al*, 1991).

Most of the trials started after the Consensus Conference, adopted the regimen 5FU+LEV as the standard arm; however, such strategy was never substantiated by the evidence of additive effects of the two drugs, or by the activity of the combination in the metastatic setting. Consequently, it was felt that more evidence was needed to evaluate the effect of this combination.

On the other hand, it was known that biomodulation of 5FU with folinic acid (FA), significantly increased response rate in advanced colorectal cancer (Advanced Colorectal Cancer Meta-Analysis Project, 1992), when compared with single agents 5FU, even though no clear evidence was available on survival.

In this contest of knowledge, in 1991, we began a trial to evaluate the independent role of LEV and FA, when combined with 5FU, in the adjuvant treatment of colorectal cancer patients. In order to reduce the possible confounding variables, we adopted the schedule of 5FU suggested by the INT-0035 trial and we decided to maintain it either in the arm of 5FU alone or in the arms of 5FU plus LEV and/or FA.

## MATERIALS AND METHODS

### Study design and statistical methods

The randomised multicentre Inter-Group Centro-Sud in COLorectal cancer (IGCS-COL) study was a 2 × 2 factorial trial addressing two questions: (1) efficacy of addition of LEV and (2) efficacy of addition of FA, to 5FU chemotherapy. Subjects were randomly assigned to one of four treatment arms (5FU alone, 5FU+LEV, 5FU+FA, 5FU+LEV+FA). IGCS-COL derived, in September 1993, from the combination of two previous independent multicentre trials, both started in 1991, and both funded by the Italian National Research Council (CNR). The first study was coordinated by the Gruppo Oncologico Centro Sud Isole – Gruppo Cooperativo Oncologico Siciliano (GOCSI-GruCOS), and the second one by the NCI in Rome – Institute Regina Elena (IRE). Decision of merging the two trials was prompted by the scientific committee of the funding agency because of the lower than expected enrolment rate and the factorial design of the two studies, that shared the question on FA. Main characteristics of the three studies are reported in Table 1.

From the start, the GOCSI-GruCOS trial addressed both the questions on LEV and FA, but chemotherapy administration was planned for 1 year. Centralised randomisation was stratified by tumour stage (II, III) and site (colon, rectum).

Conversely, together with the evaluation of FA, the IRE trial addressed the question of the efficacy of the addition of chemotherapy (with mitomycin C+5FU) given through portal vein infusion to systemic treatment given for 6 months; patients with rectal cancer were not eligible. Centralised randomisation was stratified by centre.

In September 1993, the two trials were joined under the nickname IGCS-COL (Inter-Gruppo Centro Sud – COLorectal) with a common amended protocol with the following characteristics: (a) the study had a factorial design with two questions that addressed the role of LEV and the role of FA, with four treatment arms (5FU alone, 5FU+LEV, 5FU+FA, 5FU+LEV+FA); (b) the duration of chemotherapy was established to 6 months in all arms; (c) patients with stage II or III colon or rectal cancer were eligible; (d) stage and tumour site were used as strata for randomisation. The portal vein infusion arms of the IRE trial were closed and are not accounted for in this analysis.

Randomisation was centralised at the two data centres, in Naples for GOCSI-GRUCOS centres and in Rome for IRE centres. Treatment allocation used random permuted blocks of variable size within four strata built with tumour stage (II or III) and site (colon or rectum).

With 5% type I error and 80% power of detecting a hazard ratio (HR) of relapse of 0.75 (from 50 to 60% DFS at 5 years), an expected enrolment duration of 7 years and a mean rate of 15 patients per month, 1250 patients had to be recruited and 383 events had to be observed. For the same HR to be detected in overall survival (from 60 to 68% OAS at 5 years), 314 events were needed.

Analyses were conducted on an intention-to-treat basis. DFS was defined as the interval elapsed between date of randomisation and date of the first tumour relapse (local or distant recurrence) or the date of death without relapse. Patients who were diagnosed a second primary during follow-up were censored at the date of such diagnosis. Overall survival was defined as the interval between date of randomisation and the date of death for any cause or the date of the last information on vital status.

Although results were reported for the four treatment arms, only two comparisons were planned, for FA and LEV efficacy, respectively. Survival curves were drawn by the Kaplan–Meier method and statistical significance of the differences was calculated by log-rank test. Cox multivariable regression model was adjusted by major prognostic factors (sex, stage, site of primary, grade of differentiation) stratified by site of coordination (Naples or Rome) and period of study (before or after fusion). HRs of relapse and death were estimated with 95% confidence intervals (CI). All *P-*values are two sided. Toxicity grades were compared by means of exact Wilcoxon–Mann–Whitney test.

Analyses were performed with the S-PLUS 2000 Professional statistical software package (MathSoft Inc., Seattle, WA, USA) and the StatXact-5 software (Cytel Software Corporation).

### Patients

To be eligible the patients had to meet the following criteria: histological proof of radically resected (no evidence of gross or microscopic residual disease) colon or rectal cancer; TNM stage II or III; age 18–75 years; ECOG Performance Status not worse than 2; no previous malignancy or chemotherapy; blood, liver, renal and cardiac function within normal ranges. All patients had to give oral consent to randomisation.

### Treatments

The schedule of 5FU administration was mutuated from the Intergroup trial (Laurie *et al*, 1989) for all the four treatment arms: (a) 5FU 450 mg m^−2^ i.v. bolus from days 1 to 5, then, from day 28, once weekly, for 6 months; (b) 5FU 450 mg m^−2^ i.v. bolus from days 1 to 5, then, from day 28, once weekly, for 6 months+LEV 150 mg die^−1^ orally for 3 consecutive days every 2 weeks for 6 months; (c) 5FU 450 mg m^−2^ i.v. bolus from days 1 to 5, then, from day 28, once weekly, for 6 months+FA (L-isomer) 100 mg m^−2^ i.v. 90–120 min infusion before 5FU administration; (d) 5FU 450 mg m^−2^ i.v. bolus from days 1 to 5, then, from day 28, once weekly+LEV 150 mg die^−1^ orally for 3 consecutive days every 2 weeks+FA (L-isomer) 100 mg m^−2^ i.v. 90–120 min infusion before 5FU administration, for 6 months. The dose of FA was doubled (200 mg m^−2^) if the racemic (DL) isomer was used.

For rectal cancer patients, external pelvic radiation therapy (1.8 Gy day^−1^ for 5 days week^−1^ for 5 weeks, i.e. a total dose of 45 Gy) was planned after surgery.

### Toxicity assessment

Toxicity was assessed at each chemotherapy administration, and codified according to WHO criteria (Miller *et al*, 1981). The dose of 5FU was modified depending on the type and the severity of adverse events. General rules of dose modification were: (a) in case of haematological toxicity, chemotherapy was held for a week, and resumed with the full dose if toxicity was grade 0, or with a reduced dose in case of persistent toxicity (75% of the initial dose with grade 1 or 50% of the initial dose with grade 2); (b) in case of nonhaematological toxicity, chemotherapy was held until complete resolution of the adverse event, for a maximum of 3 weeks; after 3 weeks, if toxicity was still present, the treatment was discontinued.

### Follow-up

All patients were followed every 3 months during the first 2 years, every 6 months from years 3 to 10, and annually thereafter. The follow-up evaluation included: physical examination, complete haematology and chemistry every 6 months, colonoscopy, liver ultrasound, and chest X-rays yearly.

## RESULTS

### Patients characteristics

**Figure 1:**
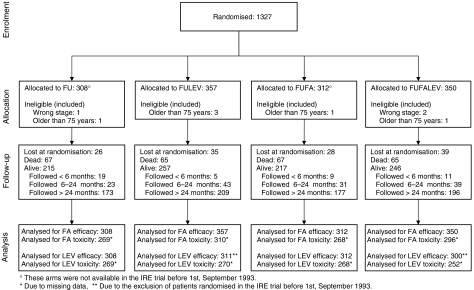
Study flow according to CONSORT.

From March 1991, to September 1998, 1327 patients were assigned 5FU alone (308 patients, 23.2%), 5FU+LEV (357 patients, 26.9%), 5FU+FA (312 patients, 23.5%) or 5FU+LEV+FA (350 patients, 26.4%). Nine patients were found ineligible after randomisation because of age (6 patients) or wrong stage (3 patients), but were included in the analysis (Figure 1). Overall 80% of patients had colon cancer and 20% rectal cancer; patients' characteristics by treatment arm are shown in Table 2.

### Efficacy

By the end of 2003, 397 patients relapsed and 264 patients died. Details of outcome events by treatment arm are reported in Table 3. The number and the site of first loco-regional relapse or distant metastases and the number of deaths were similar among the four treatment arms. Overall, 26 (2.0%) second primary tumours (12 colorectal, four breast, three lung, two prostate, two thyroid, one thymoma, one bladder, one leukaemia) were diagnosed during follow-up, with a similar distribution among the arms.

Neither the addition of LEV, nor the addition of FA, to 5FU, significantly affected DFS and OAS, at univariable and multivariable analyses. For patients receiving LEV, the HR of relapse was 0.99 (95% CI 0.81–1.21) and that of death was 0.94 (95% CI 0.73–1.20) at multivariable analysis. For patients receiving FA, the HR of relapse was 0.89 (95% CI 0.73–1.09) and that of death was 1.02 (95% 0.80–1.30) at multivariable analysis. Estimated DFS was 71 and 63% for patients receiving LEV compared with 71 and 64% for those not receiving LEV, at 3 and 5 years, respectively. Estimated DFS was 73 and 65% for patients receiving FA compared with 71 and 64% for those not receiving FA, at 3 and 5 years, respectively.

**Figure 2:**
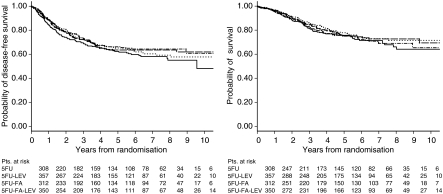
Disease-free (left) and overall survival (right) curves by treatment arms (solid=5FU, dotted=5FU+LEV, dotted/dashed=5FU+FA, dashed=5FU+LEV+FA).

DFS and OAS curves, scattered by the four treatment arms, are reported in Figure 2.

**Figure 3:**
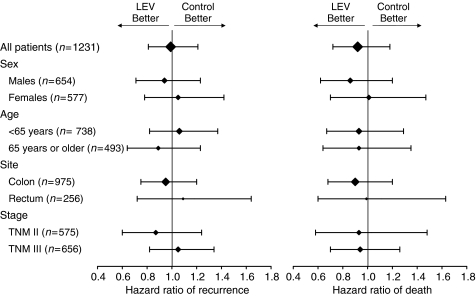
Unadjusted hazard ratio of relapse (left) and death (right) for patients receiving LEV *vs* those not receiving it. Horizontal bars represent 95% CI; size of the diamond is proportional to the size of the subgroup.

**Figure 4:**
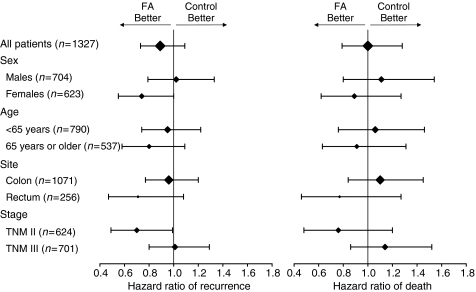
Unadjusted hazard ratio of relapse (left) and death (right) for patients receiving FA *vs* those not receiving it. Horizontal bars represent 95% CI; size of the diamond is proportional to the size of the subgroup.

Unadjusted HRs from an exploratory subgroups analysis are reported in Figures 3 and 4 for LEV and FA, respectively. No effect of LEV was observed in any subgroup; an apparent heterogeneity of FA effect on DFS was observed according to sex and stage, with higher efficacy for female and stage II patients, but for both factors the interaction tests were not statistically significant (*P*=0.09 and 0.10, respectively).

### Toxicity

Overall 13 toxic deaths (1%) were reported, with no significant difference among treatments. Toxicity, scattered by the four treatment arms, is summarised in Table 4. The addition of FA significantly worsened vomiting, diarrhoea, fever, mucositis, alopecia, skin and ocular toxicity. On the contrary, LEV did not produce any statistically significant effect on pattern of toxicity.

## DISCUSSION

In the present trial, we found no evidence of DFS or OAS improvement by adding LEV or FA to 5FU in the adjuvant treatment of patients with stage II or III colon or rectal cancer. Conversely, the addition of FA substantially increased toxicity.

As many trials published or planned at the beginning of 1990s, we pooled together patients with colon and rectal cancer (Panettiere *et al*, 1988; Laurie *et al*, 1989; Quasar Collaborative Group, 2000; Taal *et al*, 2001) and stage II or III disease (Laurie *et al*, 1989; Moertel *et al*, 1990; Wolmark *et al* 1993, 1999; Quasar Collaborative roup, 2000; Di Costanzo *et al*, 2003; Andrè *et al*, 2004). Exploratory subgroup analyses suggest that no significant heterogeneity existed in the effect of both LEV and FA across subgroups that could represent clinically relevant populations. This means that the primary results of the analysis hold true for almost all of the subgroups; although a positive effect seems evident for FA in reducing the hazard of relapse in stage II patients, such figures must be considered with caution because of the reduced sample size typical of subgroup analyses and of the obvious increased risk of false-positive results with multiple testing. However, it is important to stress that about 90% of patients with stage II colon cancer entered in this trial met criteria recently proposed by ASCO to select stage II patients candidate to adjuvant chemotherapy (Benson AB III *et al*, 2004).

5FU is still the mainstay of adjuvant treatment of colorectal cancer. Thus, the question of possible efficacy of its modulation is still actual.

Our data, that LEV does not significantly modify efficacy as well as toxicity of 5FU, are consistent with several other studies that did not find any improvement by adding LEV to 5FU both in colon (Wolmark *et al*, 1999; Quasar Collaborative Group, 2000; Cascinu *et al*, 2003) and in rectal cancer patients (Tepper *et al*, 2002); indeed, 5FU+LEV has been abandoned in clinical practice and is no more considered as a standard arm in clinical trials.

On the contrary, FA is still considered as a part of treatment schedules that include 5FU, in all ongoing trials with new drugs. In the MOSAIC study, the combination of 5FU+FA combined with oxaliplatin has shown higher efficacy than 5FU+FA alone, with a 5.3% reduction of the probability of recurrence at 3 years (Andrè *et al*, 2004). Trials adding irinotecan to 5FU+FA are ongoing and efficacy data are expected.

Our data are consistent with the results of many randomised trials (O'Connell *et al*, 1998; Bleeker *et al*, 2000; Di Costanzo *et al*, 2003) exploring the efficacy of the addition of FA to a 5FU-based regimen as adjuvant chemotherapy for colon cancer patients, which failed to demonstrate any benefit from the addition of FA. On the other hand, few studies showed the superiority of the combination 5FU+FA in terms of DFS or OAS when compared to 5FU+LEV (Wolmark *et al*, 1999; Arkenau *et al*, 2003). Overall, such contrasting data are consistent with the hypothesis that the effect of FA on efficacy, if any, is small, while the worsening effect on gastrointestinal toxicity is a common finding, well described in most papers (O'Connell *et al*, 1998; Bleeker *et al*, 2000; Porschen *et al*, 2001; Di Costanzo *et al*, 2003).

Although our study was not powered to detect any difference in the subgroup of rectal cancer patients, our finding that the addition of FA or LEV to 5FU produces no benefit, together with the results of the Intergroup-0114 trial (Tepper *et al*, 2002) and with the early report of the Intergroup-144 study (Smalley *et al*, 2003) reinforce the evidence that 5FU modulation is ineffective in rectal cancer.

On these grounds, it should be discussed whether such a small benefit may be considered still worth with schedules including new drugs (i.e. oxaliplatin or irinotecan) that may produce improvement of efficacy at a larger extent than that hypothesised for FA; in addition, with such schedules, worsening of toxicity induced by FA may become more critical for patients management, and ultimately prevent administration at full dose of more active drugs.

Unfortunately, there are no ongoing trials that add new drugs to 5FU alone. Indirect evidence could be mutuated by results recently appearing on clinical trials with capecitabine. This drug mimics a continuous infusion of 5FU, and is given without any modulating agent. The recently published results of the X-ACT trial showed a slightly significant advantage in terms of 3 years risk of relapse (HR=0.87, CI 0.75–1.00) and of death (HR=0.84, CI 0.69–1.01) for patients treated with adjuvant capecitabine with respect to those treated with standard 5FU-FA (Twelves *et al*, 2005). Safety profile, for both gastrointestinal and haematological side effects, was improved with capecitabine (Scheithauer *et al*, 2003). Such data, together with a randomised phase II trial of capecitabine in metastatic colorectal cancer suggesting that the addition of FA does not improve activity but worsen toxicity (Van Cutsem *et al*, 2000), support the hypothesis that modulation with FA might not be necessary and future trials should take into consideration the possibility of removing it from standard regimens.

## Figures and Tables

**Table 1:** Synopsis of the main characteristics of the trials before and after merging

	**IRE trial**	**GOCSI-GruCOS trial**	**IGCS-COL trial**
Main questions	(1) Efficacy of adjuvant PVI	(1) Efficacy of 5FU modulation with LEV	(1) Efficacy of 5FU modulation with LEV
	(2) Efficacy of 5FU modulation with FA	(2) Efficacy of 5FU modulation with FA	(2) Efficacy of 5FU modulation with FA
			
Patients included	Colon cancer, TNM stage II or III; age 18–75 years; PS 0–2	Colon or rectal cancer, TNM stage II or III; age 18–75 years; PS 0–2	Colon or rectal cancer, TNM stage II or III; age 18–75 years; PS 0–2
			
Study design	Phase 3, 2 × 2 factorial design	Phase 3, 2 × 2 factorial design	Phase 3, 2 × 2 factorial design
			
Portal vein chemotherapy	Mitomycin C+5FU	None	None
			
Systemic chemotherapy	5FU+LEV	5FU	5FU
	5FU+LEV+PVI	5FU+LEV	5FU+LEV
	5FU+LEV+FA	5FU+FA	5FU+FA
	5FU+LEV+FA+PVI	5FU+LEV+FA	5FU+LEV+FA
			
Randomisation	Centralised, during surgery, stratified by centre	Centralised, after surgery, stratified by tumour site and stage	Centralised, after surgery, stratified by tumour site and stage
			
Duration of systemic chemotherapy	6 months	12 months	6 months
			
Timing of follow-up	Every 3 months for years 1–2, every 6 months for years 3–10, annually thereafter	Every 3 months for years 1–2, every 6 months for years 3–10, annually thereafter	Every 3 months for years 1–2, every 6 months for years 3–10, annually thereafter
			
Time of accrual	March 1991–August 1993	March 1991–August 1993	September 1993–1998

5FU=5-fluorouracil; LEV=levamisol; PVI=portal vein infusion; PS=performance status.

**Table 2:** Characteristics of patients

**Variable**	**5FU** ***n*=308**	**5FU-LEV** ***n*=357**	**5FU-FA** ***n*=312**	**5FU-FA-LEV** ***n*=350**	**Total** ***n*=1327**
Median age (range), years	61 (22–76)	61 (26–78)	61 (26–76)	60 (25–76)	61 (22–78)
					
Gender, n (%)					
Male	157 (51)	173 (48)	164 (53)	210 (60)	704 (53)
Female	151 (49)	184 (52)	148 (47)	140 (40)	623 (47)
					
Site, n (%)					
Colon	242 (79)	292 (82)	245 (79)	292 (83)	1071 (81)
Rectum	66 (21)	65 (18)	67 (21)	58 (17)	256 (19)
					
AJCC/UICC stage, n (%)					
II	144 (47)	166 (46)	145 (46)	169 (48)	624 (47)
III	164 (53)	190 (53)	167 (54)	180 (51)	701 (53)
Unknown	—	1 (<1)	—	1 (<1)	2 (<1)
					
Tumour type, n (%)					
Adenocarcinoma	252 (82)	286 (80)	256 (82)	278 (79)	1072 (81)
Mucinous	23 (7)	30 (8)	19 (6)	27 (8)	99 (7)
Other	5 (2)	2 (1)	3 (1)	4 (1)	14 (1)
Unknown	28 (9)	39 (11)	34 (11)	41 (12)	142 (11)
					
Tumour grade, n (%)					
Well differentiated	18 (6)	21 (6)	6 (2)	17 (5)	62 (5)
Intermediate	213 (69)	227 (64)	216 (69)	237 (68)	893 (67)
Poorly differentiated	41 (13)	52 (15)	44 (14)	49 (14)	186 (14)
Unknown	36 (12)	57 (16)	46 (15)	47 (13)	186 (14)
					
Site and period of trial coordination, n (%)					
Naples, before fusion	56 (18)	56 (16)	54 (17)	56 (16)	222 (17)
Rome, before fusion	—	46 (13)	—	50 (14)	96 (7)
Naples, after fusion	202 (66)	195 (55)	200 (64)	197 (56)	794 (60)
Rome, after fusion	50 (16)	60 (17)	58 (19)	47 (13)	215 (16)

**Table 3:** Description of events by treatment arm

**Type of event**	**5FU** ***n*=308**	**5FU-LEV** ***n*=357**	**5FU-FA** ***n*=312**	**5FU-FA-LEV** ***n*=350**	**Total** ***n*=1327**
First event, *n* (%)					
Locoregional relapse					
Anastomosis or perianastomosis	5 (1.6)	2 (0.6)	2 (0.6)	5 (1.4)	14 (1.1)
Abdominal or pelvic lymphnodes	4 (1.2)	3 (0.8)	2 (0.6)	6 (1.7)	15 (1.1)
Abdominal or pelvic masses	17 (5.5)	22 (6.1)	15 (4.8)	17 (4.9)	71 (5.3)
Peritoneal carcinosis	7 (2.3)	4 (1.1)	2 (0.6)	4 (1.1)	17 (1.3)
Distant metastases					
Liver	28 (9.1)	32 (9.0)	27 (8.6)	31 (8.9)	118 (8.9)
Lung	7 (2.3)	8 (2.2)	9 (2.9)	4 (1.1)	28 (2.1)
Bone	2 (0.6)	2 (0.6)	1 (0.3)	2 (0.6)	7 (0.5)
Soft tissues	1 (0.3)	1 (0.3)	—	1 (0.3)	3 (0.2)
Combination of distant sites	8 (2.6)	2 (0.6)	5 (1.6)	5 (1.4)	20 (1.5)
					
Locoregional+distant	11 (3.6)	10 (2.8)	7 (2.2)	10 (2.9)	38 (2.9)
Other or unknown site	3 (1.0)	8 (2.2)	6 (1.9)	6 (1.7)	23 (1.7)
Death without relapse	10 (3.2)	13 (3.6)	12 (3.8)	8 (2.3)	43 (3.2)
Death, *n* (%)	67 (21.7)	65 (18.2)	67 (21.5)	65 (18.6)	264 (19.9)
					
Second primary, *n* (%)					
Colorectal	3 (1.0)	2 (0.6)	2 (0.6)	5 (1.4)	12 (0.9)
Leukemia	—	1 (0.3)	—	—	1 (0.07)
Breast	1 (0.3)	1 (0.3)	1 (0.3)	1 (0.3)	4 (0.3)
Lung	—	1 (0.3)	1 (0.3)	1 (0.3)	3 (0.2)
Thymoma	1 (0.3)	—	—	—	1 (0.07)
Bladder	—	1 (0.3)	—	—	1 (0.07)
Prostate	—	—	1 (0.3)	1 (0.3)	2 (0.1)
Thyroid	—	—	2 (0.6)	—	2 (0.1)

**Table 4:** Percentage of patients experiencing toxicity by treatment arm according to WHO grades

	**5FU**				**5FU-LEV**				**5FU-FA**				**5FU-FA-LEV**				***P*-value**	***P*-value**
	***n*=269**				***n*=310**				***n*=268**				***n*=296**				**FA effect** **(*n*=1143)**	**LEV effect (*n*=1059)**
**Type of toxicity**	**G1**	**G2**	**G3**	**G4**	**G1**	**G2**	**G3**	**G4**	**G1**	**G2**	**G3**	**G4**	**G1**	**G2**	**G3**	**G4**	**all grades**	**all grades**
Vomiting	15	4	0.4	—	17	7	0.3	0.6	17	8.6	1.9	0.4	22	8.4	1	0.3	0.004	0.11
Diarrhoea	13	5.9	2.2	—	12	11	3.5	—	10	19	9	1	12	15	8	1.7	<0.0001	0.56
Mucositis	8.9	3	0.3	0.3	9.7	5.8	0.9	—	16	8.2	9.7	2.2	17	13	3.7	2.7	<0.0001	0.24
Fever	—	—	—	—	—	0.3	0.3	—	1.5	0.7	0.3	—	1	1	0.3	0.3	0.0017	0.35
Leukopenia	8.6	6.3	0.7	0.3	9.4	6.8	0.9	—	9.3	5.2	1.9	0.7	8.8	5.4	0.3	1	0.92	0.77
Anemia	2.6	—	—	—	1.9	1.3	—	—	4.5	1.1	—	—	3.7	0.7	—	—	0.09	0.75
Thrombocytopenia	3.3	0.3	—	—	2.6	0.3	0.3	—	2.2	0.3	0.3	—	1	—	0.3	—	0.20	0.48
Skin	3.3	0.3	—	—	3.5	2.3	—	—	6.3	3.7	1.1	—	6.8	4.4	1	—	<0.0001	0.29
Congiuntivitis	—	—	—	—	0.6	—	—	—	1.9	1.9	—	—	3	0.7	—	—	<0.0001	0.77
Alopecia	1.5	0.3	—	—	1.3	0.6	—	—	3.4	1.1	1.1	—	2.4	3	0.3	—	0.0007	0.96
Abdominal pain	1.1	—	—	—	1.3	0.3	—	—	0.3	0.3	0.3	—	2	—	—	—	0.70	0.55
Anorexia	—	—	—	—	0.3	—	—	—	—	—	—	—	0.3	—	—	—	0.99	—
Taste alteration	0.3	—	—	—	—	—	—	—	0.3	—	—	—	—	—	—	—	0.99	0.51
Gastric ulcer	—	—	—	0.3	—	0.3	—	—	—	—	—	—	—	—	—	—	0.49	0.99
GI tract bleeding	0.3	—	0.3	—	—	—	—	—	0.3	—	—	—	—	—	—	—	0.87	0.26
Constipation	0.3	—	—	—	1	0.3	0.3	—	—	0.7	—	—	0.7	0.7	—	—	0.96	0.11
Bilirubin	1.1	0.3	—	—	0.9	0.3	—	—	0.7	0.3	—	—	1	0.3	—	—	0.96	0.99
Hypertransaminasaemia	2.2	0.3	—	—	1.6	0.3	0.3	—	1.1	—	—	—	1.4	0.3	—	—	0.22	0.75
Headhache	—	—	—	—	—	—	—	—	—	—	—	—	0.6	—	—	—	0.25	0.49
Fatigue	0.7	—	—	—	0.6	—	—	—	2.6	—	—	—	1.4	0.3	—	—	0.04	0.85
Cardiac	1.1	—	—	1.1	0.6	—	—	0.3	0.3	0.3	—	0.7	—	0.3	—	0.3	0.57	0.23
Neurotoxicity	—	—	—	—	0.3	0.3	—	0.3	0.3	—	—	1.1	0.7	—	—	0.7	0.10	0.48
Cistitis	—	—	0.3	—	0.3	0.3	—	—	0.3	—	—	—	0.7	—	—	—	0.99	0.95
Renal	0.3	—	—	—	0.6	—	—	—	0.3	—	—	—	0.7	—	—	—	0.99	0.97
Vascular	—	0.3	—	—	—	0.3	0.3	—	—	0.3	—	—	—	—	—	—	0.57	0.99
Allergy	—	—	—	—	0.3	—	—	—	—	—	—	—	0.7	—	—	—	0.61	0.24
Other	—	—	—	—	0.6	—	—	—	0.3	0.3	—	—	1.4	—	—	—	0.14	0.43
Toxic death	4 (1.5 %)				1 (0.3 %)				5 (1.9 %)				3 (1.0 %)				0.42	0.26

## Appendix

### Complete list of all the investigators of the centres referring to GOCSI-GruCOS cooperative groups

Oncologia Medica, Università Federico II, Napoli (A Raffaele Bianco, Sabino De Placido, Chiara Carlomagno, Rossella Lauria, Alfredo Marinelli, Amalia Milano, Clorindo Pagliarulo, Stefano Pepe, Giampaolo Tortora); Ospedale Mariano Santo, Cosenza (Salvatore Palazzo, Virginia Liguori, Candida Mastroianni, Antonio Rovito, Rosalbino Biamonte); Ospedale San Carlo, Potenza (Luigi Manzione, Domenico Bilancia, Gerardo Rosati); Ospedale Moscati, Avellino (Carlo Iannace, Francesco Caracciolo); Oncologia Medica, Benevento (Giovanni Pietro Ianniello, Vincenza Tinessa, Angela Ruggiero, Luigi Leparulo, Ghassan Merkabaoui); Oncologia Medica, Seconda Università, Napoli (Giuseppe Catalano, Fernando De Vita, Michele Orditura, Erika Martinelli, Floriana Morgillo); Oncologia Medica, Università de L'Aquila (Corrado Ficorella, Paolo Marchetti); Oncologia Medica, Università, Sassari (Antonio Farris, Giuseppina Sarobba, Gianni Sanna, M Grazia Alicicco, Carlo Potzu); Ospedale SM Goretti, Latina (Modesto D'Aprile, Giorgio Pistillucci, Franco Angelici, Venerina Sciacca); Dipartimento di Oncologia Medica, Policlinico, Messina (Vincenzo Adamo, Giuseppe Altavilla, Rosalba Rossello, Claudia Garipoli); U.O. di Oncologia Medica, Università Palermo (Nicola Gebbia, Giuseppe Cicero, Fabio Fulfaro, M Rosaria Valerio); Cattedra di Oncologia Medica, Università Palermo (Sergio Palmeri, Carlo Arcara, Giuseppe Badalamenti, Marina Vaglica); Oncologia Preventiva, Istituto Regina Elena, Roma (Silverio Tomao, Adriana Romiti); Ospedale Santi Currò, Catania (Roberto Bordonaro, Giuseppe Failla); Oncologia Medica, Università, Cagliari (Bruno Massidda, M Teresa Ionta); Oncologia Medica A, Istituto Nazionale di Tumori, Napoli (Giuseppe Comella, Rossana Casaretti); Ospedale Fatebenefratelli, Benevento (Tonino Pedicini, Antonio Febbraro); Ospedale Civile Lamezia Terme (Ettore Greco); Clinica Oncologica, Università G D'Annunzio, Chieti (Stefano Iacobelli, Luciana Irtelli, M Teresa Martino); Oncologia Medica A, Istituto Regina Elena, Roma (Francesco Cognetti); Oncologia Medica, II Facoltà, Università La Sapienza, Roma (Salvatore Lauro); Oncologia Medica B, Istituto Nazionale di Tumori, Napoli (Giovanni Salvatore Bruni); Istituto Nazionale Tumori, Bari (Vito Lorusso); Oncologia Medica, Università, Siena (Guido Francini); Oncologia Medica, Università, Cagliari (Giovanni Mantovani); Chirurgia Generale, Università Federico II, Napoli (Giovanni Persico, Andrea Renda, Luigi Bucci).

### Complete list of all the investigators of the centres referring to IRE cooperative group

Oncologia Medica B, Istituto Nazionale Tumori ‘Regina Elena’, Roma (Massimo Lopez, Giancarlo Paoletti, Luigi Di Lauro, Antonella Amodio, Paolo Foggi, Carolina Cauchi); Chirurgia Addominale, Istituto Nazionale Tumori ‘Regina Elena’, Roma (Eugenio Santoro, Maurizio Cosimelli, Fabio Carboni); Chirurgia Generale, Istituto Nazionale Tumori ‘Regina Elena’, Roma (Francesco Cavaliere, Pasquale Perri); U.O. Complessa Oncologia ‘B’, Università La Sapienza, Roma (Enrico Cortesi, Giuliana D'Auria, Adolfo De Pasquale Ceratti, Maria Laura Evangelista); Chirurgia Generale, Ospedale ‘Dono Svizzero’, Formia (Giuseppe Cardi, Alessandro Sparagna); Oncologia, Ospedale Belcolle, Viterbo (Massimo Gemini, Domenico Padalino, Carlo Signorelli, Elisabetta Capomolla); Chirurgia Generale, Ospedale Nuovo Regina Margherita, Roma (Francesco Paolo Pantano, Carlo Tirelli); Chirurgia Generale ‘Flajani’, Azienda Ospedaliera S Camillo-Forlanini, Roma (Roberto Tersigni); Chirurgia Generale, Ospedale Civile C Bernardini, Palestrina (Salvatore Naclerio); Chirurgia Vascolare, Azienda Ospedaliera Universitaria Pisana (Savino Gerardo Sardella); Oncologia Medica e Sperimentale, Istituto Oncologico di Bari (Giuseppe Colucci).
